# Supplementary material for: The Systems Biology Research Tool: evolvable open-source software
Source: BMC Syst Biol. 2008 Jun 29;2:55. doi: 10.1186/1752-0509-2-55 (PMC2446383; doi:10.1186/1752-0509-2-55)
Supplement: Additional file 1 — SBRT Archive. An archive of the current version of the Systems Biology Research Tool. [file 1752-0509-2-55-S1.zip › sbrt-1.4.0/doc/developers_guide/api/sbrt/kernel/utilities/proc/IntervalComparison.html]

IntervalComparison


|  |  |  |
| --- | --- | --- |
| **Overview** \| **Package** \| **Class** \| **Use** \| **Tree** \| **Deprecated** \| **Index** \| **Help** | |  |
| PREV CLASS   **NEXT CLASS** | **FRAMES**    **NO FRAMES**     **All Classes** |  |
| SUMMARY: NESTED \| FIELD \| CONSTR \| METHOD | DETAIL: FIELD \| CONSTR \| METHOD |  |


---


## sbrt.kernel.utilities.proc Class IntervalComparison<V>

```
java.lang.Object
  sbrt.kernel.utilities.proc.IntervalComparison<V>
```

**Type Parameters:**: `V` - the variable type.

**All Implemented Interfaces:**: KernelProcess<java.util.Set<IntervalComparer.Diff<V>>>, MonitoredProcess<java.util.Set<IntervalComparer.Diff<V>>>

---

``` public class IntervalComparison<V> extends java.lang.Object implements MonitoredProcess<java.util.Set<IntervalComparer.Diff<V>>> ```

This class is used to compare sets of intervals.

**Author:**
:   This class was written and documented by Jeremiah Wright
    while in the Wagner lab.

---

**Constructor Summary**

`IntervalComparison()`


| **Method Summary** | |
| --- | --- |
| `ProgressMonitor` | `getProgress()`             Returns a progress monitor for this process. |
| `double` | `getTolerance()` |
| `void` | `run(ResultsManager<java.util.Set<IntervalComparer.Diff<V>>> resultsManager)`             Adds to the provided results manager the variables (keys) whose intervals (values) are unequal within the provided tolerance. |
| `void` | `setIntervalSources(java.lang.Iterable<java.util.Map<V,Interval>> source1, java.lang.Iterable<java.util.Map<V,Interval>> source2)` |
| `void` | `setTolerance(double tolerance)` |

**Methods inherited from class java.lang.Object**

`clone, equals, finalize, getClass, hashCode, notify, notifyAll, toString, wait, wait, wait`

**Constructor Detail**

### IntervalComparison

```
public IntervalComparison()
```


**Method Detail**

### setTolerance

```
public void setTolerance(double tolerance)
```

---


### getTolerance

```
public double getTolerance()
```

---


### setIntervalSources

```
public void setIntervalSources(java.lang.Iterable<java.util.Map<V,Interval>> source1,
                               java.lang.Iterable<java.util.Map<V,Interval>> source2)
```

---


### getProgress

```
public ProgressMonitor getProgress()
```

:   **Description copied from interface: `MonitoredProcess`**
:   Returns a progress monitor for this process.

    :   **Specified by:**: `getProgress` in interface `MonitoredProcess<java.util.Set<IntervalComparer.Diff<V>>>`
    :   **Returns:**: a progress monitor for this process.

---


### run

```
public void run(ResultsManager<java.util.Set<IntervalComparer.Diff<V>>> resultsManager)
```

:   Adds to the provided results manager the variables (keys) whose intervals
    (values) are unequal within the provided tolerance.

    :   **Specified by:**: `run` in interface `KernelProcess<java.util.Set<IntervalComparer.Diff<V>>>`
    :   **Parameters:**: `resultsManager` - the results manager to sets of variables are added.


---


|  |  |  |
| --- | --- | --- |
| **Overview** \| **Package** \| **Class** \| **Use** \| **Tree** \| **Deprecated** \| **Index** \| **Help** | |  |
| PREV CLASS   **NEXT CLASS** | **FRAMES**    **NO FRAMES**     **All Classes** |  |
| SUMMARY: NESTED \| FIELD \| CONSTR \| METHOD | DETAIL: FIELD \| CONSTR \| METHOD |  |


---
